# Doppler ultrasound gating for adult cardiovascular magnetic resonance: Initial experience

**DOI:** 10.1016/j.jocmr.2025.101862

**Published:** 2025-02-13

**Authors:** Lucia D. Beissel, Fabian Kording, Christian Ruprecht, Alexander Isaak, Thomas M. Vollbrecht, Claus C. Pieper, Daniel Kuetting, Abdulamir Ali, Pia Wölfl, Christopher Hart, Julian A. Luetkens

**Affiliations:** a Department of Diagnostic and Interventional Radiology, University Hospital Bonn, Bonn, Germany; b Quantitative Imaging Laboratory Bonn (QILaB), University Hospital Bonn, Bonn, Germany; c Northh Medical GmbH, Hamburg, Germany; d Department of Pediatric Cardiology, University Hospital Bonn, Bonn, Germany

**Keywords:** Cardiovascular magnetic resonance, Cardiovascular magnetic resonance gating, Doppler ultrasound, Doppler ultrasound gating, Trigger delay, Trigger jitter

## Abstract

**Background:**

Despite being a common gating method for cardiovascular magnetic resonance (CMR), electrocardiogram (ECG) gating has its disadvantages, and new gating strategies are desirable. An alternative CMR gating method is Doppler ultrasound (DUS) gating, which detects blood flow and ventricular movement. The aim of this study was to prove the feasibility of DUS gating as a novel CMR gating method in a clinical patient population.

**Methods:**

In this prospective study, patients underwent clinically indicated CMR. Balanced steady-state free precession two-dimensional cine sequences in short-axis and 4-chamber views were acquired using ECG and DUS gating. DUS and ECG signal were recorded simultaneously. Time difference between R-wave and DUS systolic trigger detection was defined as trigger delay, the standard deviation of trigger delays as trigger jitter. Left and right ventricular parameters were assessed: left and right ventricular ejection fraction (LVEF, RVEF) and left and right ventricular end-diastolic volume index (LVEDVI, RVEDVI). Overall image quality was assessed using a 5-point Likert scale (5 = excellent to 1 = non-diagnostic). For statistical analysis, paired *t*-test, Wilcoxon test, Pearson correlation, and intraclass correlation coefficient (ICC) were employed.

**Results:**

Twenty-one patients (7 female) were included (age: 45.4 ± 19.7 years; body mass index: 27.6 ± 5.5 kg/m^2^). DUS mean trigger delay was 128 ± 28 ms. DUS mean trigger jitter was 23 ± 13 ms. Overall image quality showed no difference between ECG and DUS gating (e.g., short axis: 5 [interquartile range (IQR) 3–5] vs 4 [IQR 3.5–5]; P = 0.21). Quantitative analysis revealed no differences between ECG and DUS gating: LVEF (53.2 ± 9.2% vs 52.3 ± 9.1%; P = 0.18; ICC 0.97 [95% confidence interval [CI] 0.93–0.99]), LVEDVI (84.5 ± 15.8 mL/m^2^ vs 83.3 ± 15.8 mL/m^2^; P = 0.06; ICC 0.99 [95% CI 0.98–1.00]), RVEF (52.8 ± 8.0% vs 51.6 ± 7.2%; P = 0.06; ICC 0.96 [95% CI 0.89–0.99]), and RVEDVI (80.8 ± 17.6 mL/m^2^ vs 80.9 ± 16.5 mL/m^2^; P = 0.91; ICC 0.98 [95% CI 0.96–0.99]). In one patient with a prominent lingula of the lung image quality was non-diagnostic with DUS gating.

**Conclusion:**

CMR gating with DUS is feasible and can offer an equivalent performance to ECG regarding image quality and quantitative parameter assessment.

## Graphical abstract

**Figure:**
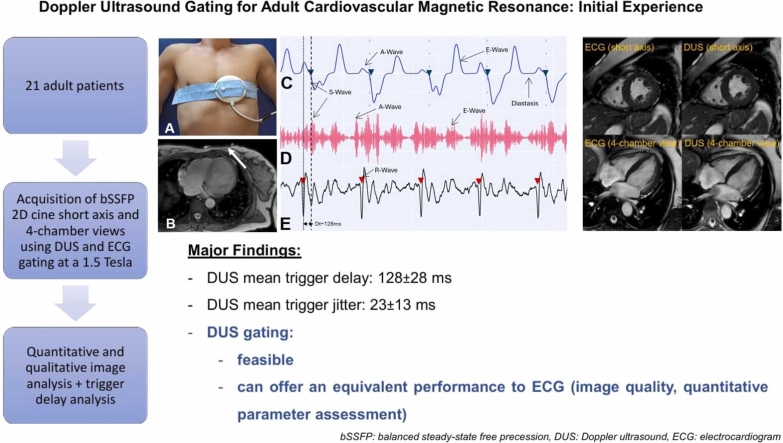

## 1 Introduction

An important pillar of cardiovascular magnetic resonance (CMR) is the synchronization of data acquisition with the cardiac cycle to obtain artifact-free images [1], [2]. The most used gating method for CMR is electrocardiogram (ECG) where the R-wave is used for gating. Nevertheless, ECG has limitations as a gating method: the need for electrodes and patient-specific parameters, such as body hair or sweating, may cause ECG-signal degradation resulting in an image quality reduction [2]. Moreover, the magnetohydrodynamic effect causes inaccuracies in the ECG, especially at higher field strengths leading to incorrect R-wave detection and reduced image quality [3], [4], [5].

Recently, cardiac gating using Doppler ultrasound (DUS) has been established as a new gating method [3], [6]. Unlike ECG, DUS detects blood flow and ventricular movement and is not susceptible to interference from the magnetic field [3], [4]. In fetal CMR, DUS gating has already evolved to a clinically valuable gating method [7], [8]. The aim of this study is to evaluate the feasibility of DUS as a gating method in an adult clinical patient population.

## 2 Materials and methods

### 2.1 Study design and participants

This prospective single-center study was approved by the institutional review board. Written informed consent was obtained from each participant.

### 2.2 Image acquisition

**Fig. 1:**
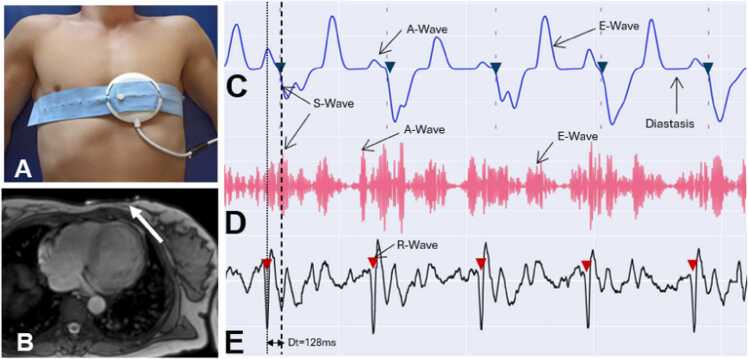
(A) Typical position of the Doppler ultrasound (DUS) transducer fixed with a belt. (B) Position of the transducer (white arrow) relative to the heart position. (C) The filtered DUS signal of patient 12. (D) The demodulated Doppler signal filtered between 60 and 100 Hz. The used S-wave for DUS triggering is marked in blue, and the R-wave is marked in red. The trigger occurs in average 128 ms after the R-wave. It can be seen that part of the delay is due to the filter delay time of the filtered signal in (D) that results in the signal shown in (C). Also indicated are the A wave which occurs during atrial contraction, the E-wave during isovolumetric contraction and the diastasis as the resting period

CMR was performed at a 1.5T scanner (Philips Ingenia 1.5T, Philips Healthcare, Best, The Netherlands) using a 16-channel torso coil with digital interface. A 1.5T–3T compatible DUS transducer (acoustic window: 6 cm) connected to a sensor box (main control unit) (smart-sync, Northh Medical GmbH, Hamburg, Germany) was placed over the patient’s heart apex (while the DUS signal was derived in real-time enabling optimal positioning), fixed with a cardiotocogram belt and optical data transmission allowed the recording of ultrasound raw data outside the magnetic resonance imaging (MRI) room (Fig. 1). The sensor box emits 1 MHz ultrasound pulses with a repetition frequency of 3.2 kHz and acoustic intensity of 4.6 W/cm^2^ to the piezoelectric element in the transducer. The DUS signal is processed from the Doppler frequencies ranging between 30 and 150 Hz. A depth optimization selects a window of interest that includes myocardial motion rejecting Doppler frequencies not sourced by myocardial motion. The acoustic window is segmented into 40 different depths. In each depth, the Doppler signal is analyzed to match the typical A-, S-, and E-wave. The Doppler filters are adjusted for each segment to optimize the match and find algorithm. The window is adaptively selected per patient in a range between 2 and 20 cm. The DUS trigger was defined by the downslope of the S-wave. For trigger transmission, the ECG wireless channel from the MRI was used. The ECG and DUS signal and the respiratory data measured with a respiratory belt were recorded outside of the MRI room simultaneously for analysis using the optical cable and the wireless transmission.

Balanced steady-state free precession (bSSFP) two-dimensional (2D) cine sequences in short axis and four-chamber view were acquired using ECG and DUS gating. Scan parameters for short-axis cine images are provided in Table 1. Breath-holding was performed at end-expiration.

**Table 1:** Scan parameters for cine images.

Parameter	Short axis	Four-chamber view
Repetition time	2.8 ms	3.0 ms
Echo time	1.4 ms	1.5 ms
Flip angle	60°	60°
Acquired voxel size	1.91 × 1.9 × 8 mm	1.9 × 1.93 × 8 mm
Reconstructed voxel size	0.98 × 0.98 × 8 mm	0.98 × 0.98 × 8 mm
Temporal resolution / shot duration @ 80 bpm	35.1 ms	36.2 ms
Reconstructed heart phases	30	30
Parallel imaging factor	3	2

*bpm* beats per minute

### 2.3 Data analysis

For data analysis, the ECG and processed DUS trigger signals during image acquisition in breath-hold were used. The R-wave was manually marked in the ECG data and assigned to a DUS systolic trigger signal. DUS trigger sensitivity was calculated based on the relation between overall detected DUS trigger points and R-waves. For each method, the RR-interval length and standard deviation were calculated. The R-wave is temporally positioned before the systolic DUS trigger, as the electrical activity of the heart precedes the myocardial contraction. The time difference between the R-wave and the DUS systolic trigger detection is defined as trigger delay and the standard deviation of the trigger delays as trigger jitter.

### 2.4 Image analysis

Left and right ventricular quantitative cardiac planimetric parameters from short-axis stacks were measured using software (Medis Suite, Medis Medical Imaging, Leiden, The Netherlands). Left and right ventricular ejection fraction (LVEF, RVEF), end-diastolic volume index (LVEDVI, RVEDVI), end-systolic volume index (LVESVI, RVESVI), and left ventricular mass index (LVMI) were assessed. Visual comparison of overall image quality was made for four-chamber view and short axis using a 5-point Likert scale (5 = excellent, 4 = good, 3 = intermediate, 2 = poor, and 1 = non-diagnostic).

### 2.5 Statistical analysis

Statistical analysis was performed using SPSS (IBM, Armonk, New York, USA, Version 27) and GraphPad Prism 10 (GraphPad Software, San Diego, California, USA, Version 10). Data are presented as mean ± standard deviation or median with interquartile range (IQR). Normality was checked with the Kolmogorov-Smirnov test. Paired Student's *t*-test or Wilcoxon signed-rank test were used for group comparisons. Pearson correlation analyzed the RR intervals of DUS and ECG. The Bland-Altman method assessed agreement and bias between DUS and ECG. Intraclass correlation coefficient (ICC, two-way mixed agreement) was used for comparison of quantitative image analysis and inter-rater agreement with ratings as follows: poor (< 0.5), moderate (0.5–0.75), good (0.75–0.9), and excellent (>0.9) [9]. P-values ≤0.05 were considered statistically significant.

## 3 Results

### 3.1 Participant characteristics

Twenty-one patients (7 female and 14 male patients; age range of 17–81 years [mean: 45.4 ± 19.7 years]; body mass index range of 20.8–41.3 kg/m^2^ [mean: 27.6 ± 5.5 kg/m^2^]) were included in this study. Table 2 summarizes the demographic data.

**Table 2:** Demographics of the included patients.

Patient	Age (y)	Sex	Indications for CMR	Main CMR diagnosis
1	30	f	Cardiac arrhythmias	Normal
2	26	m	Exclusion of structural heart disease	Normal
3	36	m	Exclusion of structural heart disease	Normal
4	59	m	Exclusion of structural heart disease	Normal
5	36	m	Exclusion of structural heart disease	Normal
6	56	m	Exclusion of structural heart disease	Normal
7	33	f	Suspicion of peripartum cardiomyopathy	Peripartum cardiomyopathy
8	81	m	Exclusion of structural heart disease	LV dilatation and hypertrophy, aortic and mitral valve insufficiency, aneurysm of the ascending aorta
9	32	m	Exclusion of structural heart disease	Normal
10	70	m	Stress CMR	No ischemia, myocardial fibrosis
11	36	m	Exclusion of structural heart disease	Normal
12	60	m	Myocarditis follow-up	Inflammatory cardiomyopathy, post-inflammatory fibrosis
13	56	f	Cardiac sarcoidosis follow-up	Cardiac sarcoidosis
14	72	m	Suspicion of HOCM	HOCM
15	73	f	Stress CMR	Normal
16	32	f	Suspicion of myocarditis	Normal
17	20	f	Stress CMR	Normal
18	17	m	Exclusion of structural heart disease	Normal
19	24	m	Pericarditis follow-up	Pericarditis
20	67	f	Myocardial viability	Post-infarct scar
21	38	m	Apical left ventricular thrombus	Post-infarct scar, no thrombus

*CMR* cardiovascular magnetic resonance, *f* female, *HOCM* hypertrophic obstructive cardiomyopathy, *LV* left ventricle, *m* male

### 3.2 Trigger analysis

**Fig. 2:**
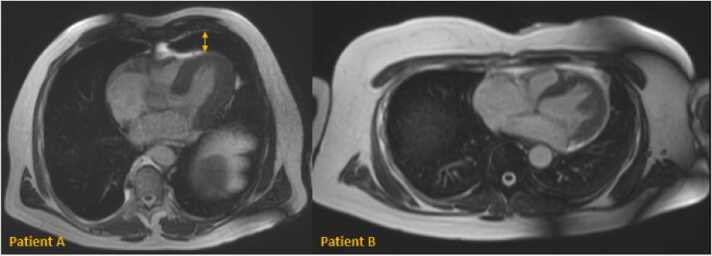
Planning sequences in two different patients (A and B). Patient A had a prominent lingula of the lung (arrow) which led to a poor acoustic window and poor trigger signal and hence to non-diagnostic images of Doppler ultrasound (DUS) gating. Patient B has optimal anatomic conditions (heart directly adjacent to the thoracic wall) for DUS gating which lead to excellent image quality

**Fig. 3:**
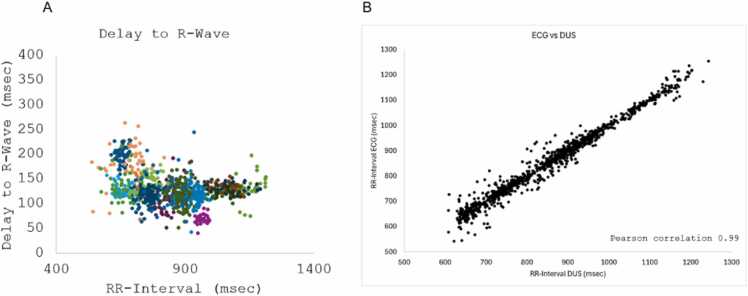
Evaluation of the Doppler ultrasound (DUS) trigger detection on simultaneously acquired ECG and DUS trigger signals. (A) Individual trigger delay of DUS to the R-wave of the ECG for each patient revealed an average trigger delay of 128 ± 28 ms. Each patient is represented by a different color. (B) Correlation between the RR-Interval of DUS and ECG. *ECG* electrocardiogram

Fig. 1 visualizes a comparison of DUS and ECG signals. DUS signal derivation was not stable in one patient with a prominent lingula of the lung leading to a poor acoustic window (Fig. 2). These data were excluded from trigger analysis and quantitative image analysis while the acquired images were included for qualitative image analysis. DUS signal derivation was uncomplicated. Table 3 illustrates the average trigger delay and trigger jitter in each patient. In Fig. 3, the trigger delay is plotted against the RR-interval.

**Table 3:** Trigger delay and trigger jitter for each patient.

Patient	#beats ECG	#beats DUS	Sensitivity (%)	Trigger delay (ms)	Trigger jitter (ms)
1	187	197	95	132	25
2	170	171	99	114	10
3	164	164	100	115	22
4	146	149	98	67	8
5	134	134	100	145	14
6	131	134	98	127	30
7	142	143	99	134	8
8	173	166	96	147	46
9	197	199	99	123	8
10	169	171	99	118	21
11	162	169	96	111	31
12*	-	-	-	-	-
13	161	161	100	120	23
14**	-	-	-	-	-
15	206	221	93	106	14
16	186	189	98	184	37
17	214	223	96	114	50
18	156	164	95	197	17
19	197	199	99	135	30
20	160	161	99	117	12
21	115	120	96	131	33
					
Average	167	170	98	128	23
STD	27	29	2	28	13

Trigger delay is defined as the time between the R-wave and the DUS systolic trigger detection. Trigger jitter is defined as the standard deviation of the trigger delays.*DUS* Doppler ultrasound, *ECG* electrocardiogram, *STD* standard deviation

* The ECG signal was incompletely recorded and could not be used for analysis

** The DUS signal could not be derived in this patient

### 3.3 Quantitative image analysis

**Fig. 4:**
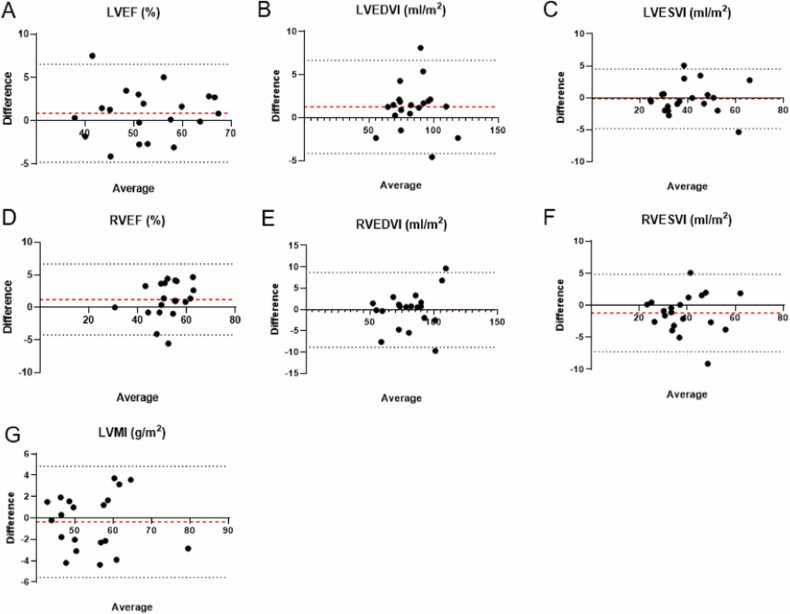
Bland-Altman plots for quantitative measurements are shown. The y-axis shows the difference in the parameters between DUS and ECG gating. The x-axis shows the mean value of measurements for both gating methods. The red dotted horizontal line shows the bias. The black dotted horizontal lines indicate the 95% confidence intervals. *LVEF* left ventricular ejection fraction, *RVEF* right ventricular ejection fraction, *LVEDVI* left ventricular end-diastolic volume index, *RVEDVI* right ventricular end-diastolic volume index, *LVESVI* left ventricular end-systolic volume index, *RVESVI* right ventricular end-systolic volume index, *LVMI* left ventricular mass index, *DUS* Doppler ultrasound, *ECG* electrocardiogram

There was no significant difference between ECG and DUS gated short-axis cine images regarding all measured planimetric data and close agreements in planimetric parameters were yielded between ECG and DUS gated images (Table 4, Fig. 4). High ICCs indicated a good agreement for the assessment of planimetric parameters. Table 4 provides an overview of the planimetric parameters and their statistical analysis.

**Table 4:** Quantitative image analysis—statistics.^a^

	Measured value with ECG gating	Measured value with DUS gating	P-value	Bland-Altman analysis		ICC
				Bias	LOA	
LVEF (%)	53.2 ± 9.2	52.3 ± 9.1	0.18	0.9 ± 2.9	−4.8 to −6.6	0.97 [95% CI 0.93–0.99]
LVEDVI (mL/m^2^)	84.5 ± 15.8	83.3 ± 15.8	0.06	1.2 ± 2.8	−4.2 to 6.7	0.99 [95% CI 0.98–1.00]
LVESVI (mL/m^2^)	39.7 ± 11.7	39.9 ± 11.6	0.44	−0.2 ± 2.4	−4.8 to 4.5	0.99 [95% CI 0.98–1.00]
LVMI (g/m^2^)	54.1 ± 8.9	54.5 ± 9.0	0.55	−0.4 ± 2.7	−5.6 to 4.8	0.98 [95% CI 0.95–1.00]
RVEF (%)	52.8 ± 8.0	51.6 ± 7.2	0.06	1.2 ± 2.8	−4.2 to 6.7	0.96 [95% CI 0.89–0.99]
RVEDVI (mL/m^2^)	80.8 ± 17.6	80.9 ± 16.5	0.91	−0.1 ± 4.5	−8.9 to 8.7	0.98 [95% CI 0.96–0.99]
RVESVI (mL/m^2^)	38.1 ± 10.4	39.3 ± 10.4	0.10	−1.2 ± 3.1	−7.2 to 4.8	0.98 [95% CI 0.94–0.99]

*CI* confidence interval, *DUS* Doppler ultrasound, *ECG* electrocardiogram, *ICC* intraclass correlation coefficient, *LOA* limits of agreement, *LVEDVI* left ventricular end-diastolic volume index, *LVEF* left ventricular ejection fraction, *LVESVI* left ventricular end-systolic volume index, *LVMI* left ventricular mass index, *RVEDVI* right ventricular end-diastolic volume index, *RVEF* right ventricular ejection fraction, *RVESVI* right ventricular end-systolic volume index

a Measurements for quantitative image analysis are presented as mean ± standard deviation.

### 3.4 Qualitative image analysis

**Fig. 5:**
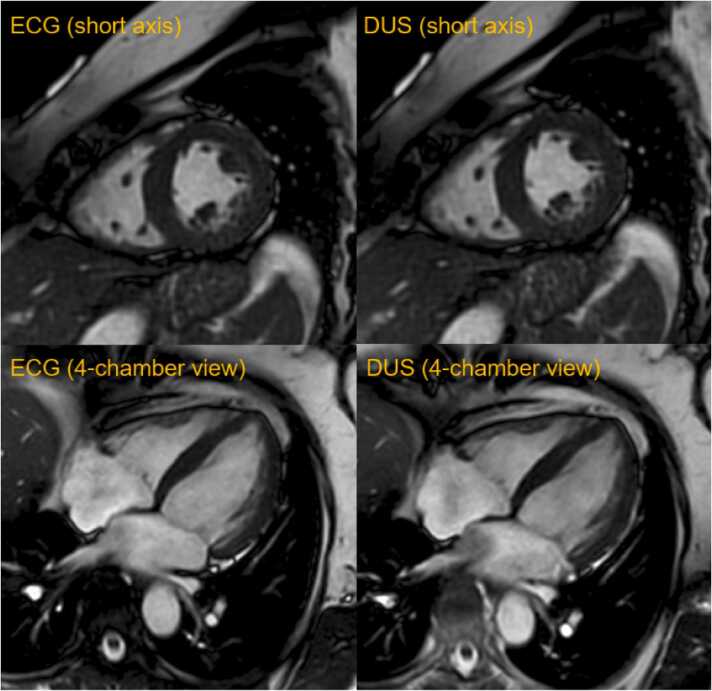
Electrocardiogram (ECG) and Doppler ultrasound (DUS) gated balanced steady-state free precession two-dimensional cine sequences in short axis and four-chamber view in a 56-year-old patient. There is no difference in overall image quality between the two gating methods

**Fig. 6:**
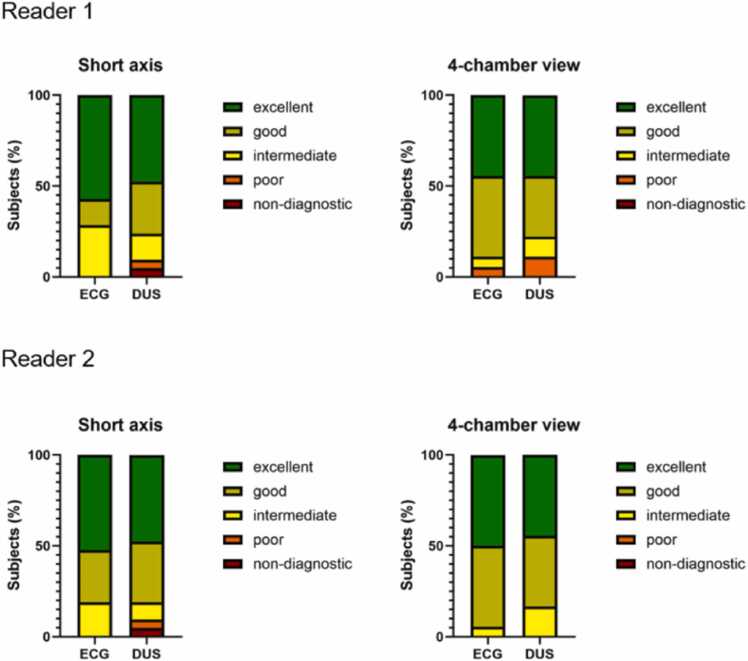
Bar plots of overall image quality ratings of electrocardiogram (ECG) and Doppler ultrasound (DUS) gated short-axis and 4-chamber view images

There was no significant difference regarding image quality between ECG and DUS gating acquired bSSFP 2D cine sequences (Fig. 5). Inter-rater agreement for qualitative image analysis was good to excellent. Fig. 6 shows the distribution of overall image quality ratings for both readers. Table 5 summarizes the statistical analysis of the qualitative image analysis.

**Table 5:** Qualitative image analysis—statistics.^a^

	Rater 1			Rater 2			ICC	
	ECG	DUS	P-Value	ECG	DUS	P-Value	ECG	DUS
Short axis	5 [IQR 3–5]	4 [IQR 3.5–5]	0.21	5 [IQR 4–5]	4 [IQR 4–5]	0.32	ICC 0.87 [95% CI 0.67–0.95]	ICC 0.97 [95% CI 0.93–0.99]
Four-chamber view	4 [IQR 4–5]	4 [IQR 3.75–5]	0.37	4.5 [IQR 4–5]	4 [IQR 4–5	0.32	ICC 0.78 [95% CI 0.42–0.92]	ICC 0.91 [95% CI 0.76–0.97]

*CI* confidence interval, *DUS* Doppler ultrasound, *ECG* electrocardiogram, *ICC* intraclass correlation coefficient, *IQR* interquartile range

a Image qualitiy ratings are presented as median and interquartile range.

## 4 Discussion

DUS-based gating, unlike ECG, is not affected by the surrounding magnetic field, therefore offering potential at high-field strengths and reduced susceptibility for set-up problems [2], [3], [4], [6], [10]. The smart-sync system was simple to operate and could be applied as quickly as an ECG. Trigger analysis revealed a trigger delay of 128 ± 28 ms between R-wave and systolic DUS trigger which was expected as electrical activity precedes the myocardial contraction. Trigger delay varied, primarily due to patient-specific factors such as slight anatomic or functional differences. Trigger jitter occurs due to variations in each cardiac cycle which can be explained by arrhythmia and hence variations in the cardiac excitation line. The trigger jitter of 23 ± 13 ms was below the temporal resolution of image acquisition.

We found an unstable DUS signal leading to non-diagnostic image quality in one patient due to a poor acoustic window. This patient had a prominent lingula between heart and chest. Anatomical variations or lung diseases can cause signal problems. Except from this single case, image quality was diagnostic in all cases.

Thoracic deformities may hinder optimal transducer placement due to an uneven thoracic wall. A cushion can help increase pressure on the transducer. Reducing the transducer size improves the likelihood of lying flat on the patient's chest. The transducer is currently about 10 cm in diameter with an acoustic window of 7 cm, and smaller transducers (2 cm) are currently being tested. Despite this, we were able to derive a stable signal in a patient with pectus excavatum.

Another advantage of DUS is the possibility to detect quiescent heart phases such as diastasis, thus permitting new opportunities in imaging of the coronary arteries due to the exact detection of the ideal gating timepoint [10]. The E-wave as trigger source provides a direct measurement of the beginning of the quiescent heart phase and is potentially more accurate than calculating the time delay from the R-wave to the quiescent phase as the beats per minute are changing over the acquisition. The key advantage of DUS gating over other gating is its direct cardiac signal derivation and ability to target specific heart phases.

## 5 Limitations

For data analysis, the ECG R-wave was manually marked and compared to the smart-sync DUS trigger points. The manual trigger detection assures 100% trigger accuracy whereas DUS trigger signals need to be calculated in almost real-time. Hence, this method can only determine the sensitivity of DUS. This might overlook potential advantages of DUS. However, this does not affect the key messages of our study.

## 6 Conclusion

DUS gating was feasible in a clinical setting, and the image quality and volumetric measurements of cine acquisitions were comparable to ECG gating. Its potentially advantageous use in high-field MRI and specific patient populations (e.g. pediatric patients or those with arrhythmia) should be further explored in larger clinical studies.

## Funding

None.

## Author contributions

**Claus C. Pieper:** Validation. **Daniel Kuetting:** Validation. **Alexander Isaak:** Validation. **Thomas M. Vollbrecht:** Validation. **Christopher Hart:** Validation. **Julian A. Luetkens:** Writing – original draft, Validation, Supervision, Resources, Project administration, Methodology, Investigation, Formal analysis, Data curation, Conceptualization. **Abdulamir Ali:** Formal analysis, Data curation. **Pia Wölfl:** Validation, Formal analysis, Data curation. **Christian Ruprecht:** Visualization, Validation, Software, Methodology, Investigation, Formal analysis, Data curation. **Lucia D. Beissel** Writing – original draft, Visualization, Project administration, Methodology, Investigation, Formal analysis, Data curation. **Fabian Kording:** Writing – original draft, Visualization, Validation, Software, Resources, Methodology, Investigation, Formal analysis, Data curation.

## Declaration of competing interests

The authors declare that they have no known competing financial interests or personal relationships that could have appeared to influence the work reported in this paper.
